# Sub‐stratification of type‐2 high airway disease for therapeutic decision‐making: A ‘bomb’ (blood eosinophils) meets ‘magnet’ (FeNO) framework

**DOI:** 10.1111/resp.14294

**Published:** 2022-05-19

**Authors:** Simon Couillard, Ian D. Pavord, Liam G. Heaney, Nayia Petousi, Timothy S. C. Hinks

**Affiliations:** ^1^ Respiratory Medicine Unit and Oxford Respiratory NIHR BRC, Nuffield Department of Medicine University of Oxford, Old Road Campus Oxford UK; ^2^ Faculté de Médecine et des Sciences de la Santé Université de Sherbrooke Sherbrooke Québec Canada; ^3^ Centre for Experimental Medicine Queen's University Belfast School of Medicine Dentistry and Biomedical Sciences Belfast UK

**Keywords:** airway markers, asthma, eosinophils, inflammation, nitric oxide

Biologic therapies targeting components of the type‐2 inflammatory response seen in many patients with obstructive airways disease have had a dramatic impact in the clinic. In the severe asthma clinic, extraordinary outcomes are being achieved^1^ and the days when the severe asthma clinicians' job was mainly about overseeing an orderly decline (much of it due to oral corticosteroid‐related morbidity) have been confined to the history books. In 2023, it is projected that sales will top $10 billion, accounting for over 30% of total worldwide asthma drug sales.^2^

The success of biologics was not predicted by all and there was a view that targeting treatment to a biomarker‐defined subgroup of patients would result in a severely restricted market.^3^ In fact, we have seen the opposite. The ability to target treatment to a population who are most likely to benefit has incentivized clinicians to look for these patients, has increased the likelihood of a good response to treatment and has resulted in low rates of treatment failure.^1^ It has also made the job of jumping regulatory hurdles and making a compelling cost–benefit case for funding of treatment much more straightforward.

We now have four monoclonal antibody targeting strategies inhibiting different aspects of the immune response driving asthma attacks (Figure 1). The target population is patients with persistent airway inflammation despite treatment with a reasonable dose of inhaled corticosteroids (ICS). The persistence of type‐2 inflammation indicates that the airway mucosal mechanisms driving recruitment of eosinophils towards the airway epithelium are, or have become, corticosteroid‐resistant. In this situation, effective treatment exploits other mechanisms to reduce eosinophilic airway inflammation: depletion of circulating eosinophils (oral corticosteroids and biologics targeting the IL‐5 pathway: mepolizumab, benralizumab, reslizumab); prevention of eosinophils leaving the vascular compartment (targeting the IL‐4 receptor and inhibiting IL‐4/IL‐13 responses: dupiliumab); or inhibition of the corticosteroid‐resistant airway mucosal drivers of type‐2 inflammation by other mechanisms (dupilumab again or targeting thymic stromal lymphopoietin [TSLP] with tezepelumab). A sixth biologic (omalizumab) targets the distal effector of the allergic reaction (IgE) with marginal effects on the type‐2 immune response.^4^

**FIGURE 1:**
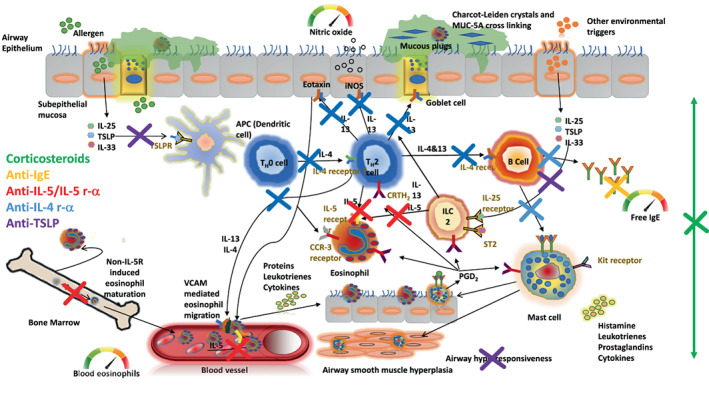
Type‐2 airway inflammation is driven by an adaptive and innate immune response driven by epithelial alarmins (particularly IL‐33 and thymic stromal lymphopoietin). Type‐2 airway inflammation is detected in clinical practice by assessing fractional exhaled nitric oxide (reflecting airway IL‐13 activity) and blood eosinophils (reflecting systemic IL‐5 activity). The main functional consequence of type‐2 inflammation is airflow limitation as a result of airway mucus plugging, airway wall oedema and thickening, airway smooth muscle hyperplasia and the induction of airway hyperresponsiveness. Key cellular players, cytokines, chemokines, effector mediators and their receptors, are shown. Coloured crosses indicate the pathways inhibited by the relevant biologic (shown in same colour); corticosteroids inhibit most pathways at the cost of associated toxicity.

The presence of multiple biologic options and the absence of any direct head‐to‐head comparisons mean that finding the right biologic for the right patient has become increasingly challenging. In this commentary article, we suggest that one way forward is to sub‐stratify type‐2 inflammatory airway disease according to the dominant driving mechanism. This concept is based on the demonstration that the two clinically accessible biomarkers of type‐2 inflammation, fractional exhaled nitric oxide (FeNO) and the blood eosinophil count, provide independent prognostic (of exacerbations) and predictive (of corticosteroid response) information because they identify different aspects of type‐2 inflammation.^5^, ^6^, ^7^, ^8^, ^9^

Mechanistic support for this idea comes from a recent cross‐sectional analysis of induced sputum and serum markers of type‐2 inflammation in patients with severe asthma who were confirmed to be adherent to high‐dose ICS. We found that FeNO correlated with almost all of the assessed components of the airway type‐2 immune response in sputum, whereas the blood eosinophil count correlated with serum IL‐5 but not with any assessed measure of airway inflammation (Figure 2).^5^ FeNO and blood eosinophils therefore relate to different components and compartments of type‐2 inflammation (Figure 2): FeNO reflects airway type‐2 activity and the chemotactic pull to the airways (the magnet), whereas blood eosinophils reflect the systemic pool of available effector cells and circulating IL‐5 (the bomb). The important clinical corollary of these mechanistic data is observable in randomized clinical trials, where raised values of baseline FeNO and blood eosinophils act synergistically to predict asthma attacks in the placebo arm^6^—an excess risk which is entirely removed by appropriate type‐2 targeting anti‐inflammatory therapy.^7^

**FIGURE 2:**
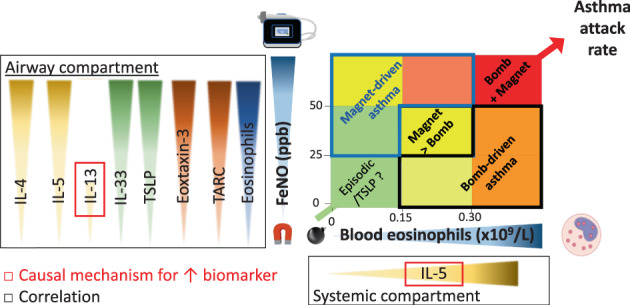
In severe asthma with documented treatment adherence to high‐dose inhaled corticosteroids (as demonstrated following a fractional exhaled nitric oxide [FeNO] suppression test), FeNO is correlated with increased induced sputum levels of airway type‐2 cytokines, chemokines and alarmins. In contrast, blood eosinophils correlate with serum IL‐5 and not with any assessed measure in sputum. These results imply that FeNO and blood eosinophils relate to different components and compartments of type‐2 inflammation, with FeNO reflecting the chemotactic pull (magnet) to the airways and blood eosinophils reflecting the systemic pool of available eosinophils (bomb). When both occur together, the risk of asthma attacks (bomb detonating) is particularly high. The biomarker profile of magnet‐driven disease is shown in the blue outlined area and bomb‐driven disease in the black outlined area overlaying a grid showing the relative risk of asthma attacks according to data from placebo arms of randomized controlled trials. Colour codes reflect the relative risk of asthma attacks: green (low risk) to red (high risk). Based on data from Couillard et al^5^, ^6^; figure reproduced from Couillard et al^10^ with permission from Elsevier.

These findings imply that we might be in a position to choose the most appropriate biologic for an individual patient based on their biomarker profile and the primary target of the biologic. Patients with a ‘magnet’‐predominant profile, that is, an FeNO‐predominant biomarker profile with ICS‐resistant inflammation, might respond best to a biologic that targets relevant airway mucosal processes such as anti‐TSLP (tezepelumab) or anti‐IL‐4 receptor alpha (dupilumab). In contrast, patients with a ‘bomb’ profile and a blood eosinophil‐predominant biomarker profile would respond best to biologics targeting IL‐5. There is some support for this concept. Shrimanker et al.^8^ showed in a post hoc analysis of the phase 3 QUEST and phase 2b DREAM study that patients with an FeNO >25 ppb but blood eosinophil count <150 cells/μl (consistent with our proposed ‘magnet’‐driven asthma profile) responded to dupilumab but not to mepolizumab. Moreover, patients with a blood eosinophil count >500 cells/μl (consistent with a ‘bomb’‐driven asthma profile) are known to have a very large and complete response to biologics targeting IL‐5, independent of FeNO.^9^, ^11^ Finally, although the anti‐TSLP tezepelumab showed efficacy in non‐type‐2 asthma,^12^ it is important to note that the greatest relative and absolute benefits were noted in populations with either FeNO or blood eosinophils raised, and that the benefits were limited to the prevention of asthma attacks but did not result in reduced oral corticosteroid dependency.^12^, ^13^ These mixed results suggest that tezepelumab protects against episodic alarmin‐mediated ‘magnet’ signalling but does not effectively disarm the effector cell (bomb) reservoir to the extent that systemic corticosteroids do.

There is an opportunity to test this hypothesis further by carrying out post hoc analyses of other biologic trials. The primary focus would be the treatment effect on asthma attack in patients stratified using a composite stratification score (combining blood eosinophils and FeNO) (Figure 2) or a nomogram weighing the strength of signal for each biomarker (Figure 3): a stronger eosinophil signal implies a ‘bomb’‐driven problem while a stronger FeNO signal identifies a ‘magnet’‐driven asthma. The expectation would be that tezepelumab and dupilumab would be more effective than biologics targeting IL‐5 in ‘magnet’‐predominant patients, whereas the opposite would be seen in ‘bomb’‐predominant patients. Additional outcome measures, such as the treatment effect on forced expiratory volume in 1 s (FEV_1_), decline in FEV_1_ and achievement of clinical remission, could be investigated with the interesting possibility that some of these outcomes would be more ‘magnet’ or ‘bomb’ associated. A final output of interest would be the other phenotypic characteristics of ‘magnet’‐ and ‘bomb’‐predominant patients. Table 1 outlines some of the differences that might be seen. We acknowledge that this bomb‐meets‐magnet therapeutic framework is largely speculative, that the two processes overlap in a significant number of patients (shown in Figure 2 but not in Figure 3 ) and that raised FeNO translates to more than just a ‘magnetic’ pull for eosinophils insofar as it relates to accelerated lung function decline. Finally, we did not include the anti‐IgE omalizumab, generally found to be less effective than the more recent type‐2‐targeting biologics.^4^

**FIGURE 3:**
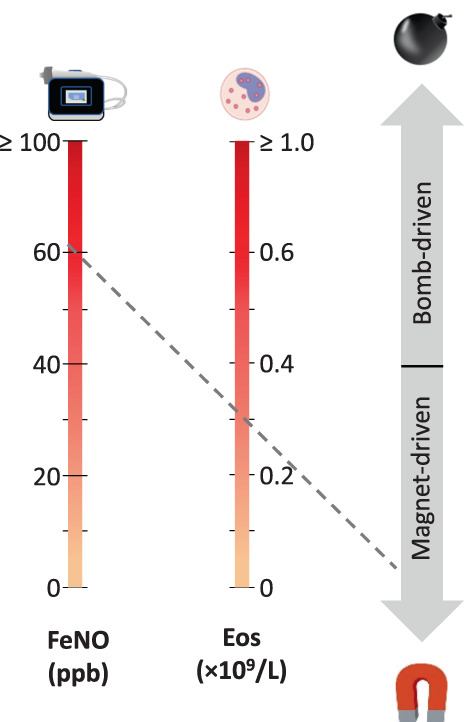
A prototype nomogram to interpret the strength of the fractional exhaled nitric oxide (FeNO) (‘magnet’) versus eosinophil (‘bomb’) signals: The line linking two biomarker values identifies bomb‐ versus magnet‐predominant asthma. Dashed grey line (—): Example of the nomogram result for a patient with an FeNO of 60 ppb and a blood eosinophil count (Eos) of 0.3 × 10^9^/L, suggesting a magnet‐driven asthma; see Table 1 for other phenotypic characteristics that may influence case interpretations. The cut‐off points and their positions on the nomogram are based on the fact that (a) clinical cut‐off points are 0.15–0.3 × 10^9^/L for blood eosinophils and 20–40 ppb for FeNO,^14^ (b) the predictive value of these biomarkers are approximately linear up 0.6 × 10^9^/L and 60 ppb, respectively, in the placebo arms of severe asthma trials^15^ and (c) upper values are logarithmically distributed.

**TABLE 1:** Potential^a^ features of ‘magnet’ and ‘bomb’ patients with type‐2 high asthma

Magnet‐driven T2 asthma	Bomb‐driven T2‐asthma
Early onset	Late onset
Allergy	No allergy
AHR++	Less AHR
Eczema, atopic rhinitis/CRSwNP	Less atopic CRSwNP, EGPA
FeNO signal > blood eos signal	Blood eos signal > FeNO signal
ICS > OCS sensitivity	OCS > ICS sensitivity
Anti‐TSLP/anti‐IL‐4Rα > anti‐IL‐5 response	Anti‐IL‐5 ~ anti‐IL‐4Rα > anti‐TSLP response

*Note*: Magnet patients have earlier onset, allergic asthma characterized by more airway hyperresponsiveness, more allergy and allergy‐associated comorbidities and a more compete response to ICS and anti‐TSLP/anti‐IL‐4Rα.

Abbreviations: AHR, airway hyperresponsiveness; CRSwNP, chronic rhinosinusitis and nasal polyposis; EGPA, eosinophilic granulomatosis and polyangiitis; eos, eosinophils; FeNO, fractional exhaled nitric oxide; ICS, inhaled corticosteroids; IL‐4Rα, IL‐4 receptor‐alpha; OCS, oral corticosteroids; TSLP, thymic stromal lymphopoietin.

^a^ The table is speculative based on the data discussed in full body.

We suggest that these post hoc studies and new comparative studies are urgently needed to test whether sub‐stratification of type‐2 high airway disease is a valid means to better match patients and biologics and determine whether this stratification allows us to make other clinically useful predictions.

## FUNDING INFORMATION

This work was supported by the Oxford Respiratory NIHR BRC.

## CONFLICTS OF INTEREST

Simon Couillard received non‐restricted research grants from Sanofi‐Genzyme, the Quebec Respiratory Health Research Network and the Association Pulmonaire du Québec, outside the submitted work; received speaker honoraria from GlaxoSmithKline, Sanofi‐Regeneron and AstraZeneca, outside the submitted work; and is on the advisory board of Biometry Inc, outside the submitted work. In the last 5 years, Ian D. Pavord has received speaker's honoraria for speaking at sponsored meetings from AstraZeneca, Boehringer Ingelheim, Aerocrine AB, Almirall, Novartis, Teva, Chiesi, Sanofi/Regeneron, Menarini and GSK, and payments for organizing educational events from AstraZeneca, GSK, Sanofi/Regeneron and Teva. He has received honoraria for attending advisory panels with Genentech, Sanofi/Regeneron, AstraZeneca, Boehringer Ingelheim, GSK, Novartis, Teva, Merck, Circassia, Chiesi and Knopp, and payments to support FDA approval meetings from GSK. He has received sponsorship to attend international scientific meetings from Boehringer Ingelheim, GSK, AstraZeneca, Teva and Chiesi. He has received grants from Chiesi and Sanofi Genzyme. He is co‐patent holder of the rights to the Leicester Cough Questionnaire and has received payments for its use in clinical trials from Merck, Bayer and Insmed. During 2014–2015, he was an expert witness for a patent dispute involving AstraZeneca and Teva. Liam G. Heaney has received grant funding, participated in advisory boards and given lectures at meetings supported by Amgen, AstraZeneca, Boehringer Ingelheim, Circassia, Hoffmann la Roche, GlaxoSmithKline, Novartis, Theravance, Evelo Biosciences, Sanofi and Teva. He has received grants from MedImmune, Novartis UK, Roche/Genentech Inc, Glaxo Smith Kline, Amgen, Genentech/Hoffman la Roche, Astra Zeneca, MedImmune, Glaxo Smith Kline, Aerocrine and Vitalograph. He has received sponsorship for attending international scientific meetings from AstraZeneca, Boehringer Ingelheim, Chiesi, GSK and Napp Pharmaceuticals. He has also taken part in asthma clinical trials sponsored by AstraZeneca, Boehringer Ingelheim, Hoffmann la Roche and GlaxoSmithKline for which his institution received remuneration. He is the Academic Lead for the Medical Research Council Stratified Medicine UK Consortium in Severe Asthma which involves industrial partnerships with a number of pharmaceutical companies including Amgen, AstraZeneca, Boehringer Ingelheim, GlaxoSmithKline, Hoffmann la Roche and Janssen. Nayia Petousi is supported from the NIHR Oxford BRC. She has received grants from the University of Oxford and NIHR research capability funding from Oxford University Hospitals outside the submitted work. She has received personal fees from Astra Zeneca, outside the submitted work. Timothy S. C. Hinks has received grants from the Wellcome Trust, The Guardians of the Beit Fellowship, Pfizer Inc., Kymab Ltd, Sensyne Health, University of Oxford and from the NIHR Oxford BRC, outside the submitted work. He has received personal fees from Astra Zeneca, TEVA, Omniprex and Peer Voice, outside the submitted work.
